# Supplementary material for: Learning curve and surgical outcome of robotic assisted colorectal surgery with ERAS program
Source: Sci Rep. 2022 Nov 29;12:20566. doi: 10.1038/s41598-022-24665-w (PMC9709162; doi:10.1038/s41598-022-24665-w)
Supplement: Supplementary file 1 — Supplementary Information 1. [file 41598_2022_24665_MOESM1_ESM.zip › LSC Raw data-Ñ[▒K/143/H_ñw╝╨░O▒Kñσ.pdf]

正式摘要(電子簽章：000000228516893)		
W83 / W83 ☐英文標題(注意:無英文姓名-請補病人基本檔!)	☐【讀取】憑證	

臺中榮民總醫院
出院病歷摘要

轉入號 : 轉出號 :

[redacted]

(8)流水編號	
(9)入院日期	110/10/24 (2021)
(10)轉科日期	
(11)出院日期	110/10/30 (2021) 住院天數計 6 日

診斷

- 入院 :1. Sigmoid colon cancer
  2. SCC of esophagus, Tm(30-35cm), cT3N1M0, s/p PEG + chemoport(2012/12/07), s/p MIE + Laparoscopic reconstruction on 2013/2/20, ypT0N0M0
  3. Thyroid nodule s/p L't thyroidectomy in 2007.
  4. HBV carrier under Entecavir 1# PO QDAC
- 出院 :1. Sigmoid colon cancer s/p laparoscopic anterior resection on 2021/10/25
  2. left pleural effusion s/p pigtail insertion on 2021/10/25, removed on 10/29
  3.SCC of esophagus, Tm(30-35cm), cT3N1M0, s/p PEG + chemoport(2012/12/07), s/p MIE + Laparoscopic reconstruction on 2013/2/20, ypT0N0M0
  4. Thyroid nodule s/p L't thyroidectomy in 2007.
  5. HBV carrier under Entecavir 1# PO QDAC

主訴

- blood-tinged stool for about 1 month

病史

- This 54 years old female patient has past history of
  1. SCC of esophagus, Tm(30-35cm), cT3N1M0, s/p PEG + chemoport(2012/12/07), s/p MIE + Laparoscopic reconstruction on 2013/2/20, ypT0N0M0
  2. Thyroid nodule s/p L't thyroidectomy in 2007.
  3. HBV carrier under Entecavir 1# PO QDAC
  She recieved regular CS OPD follow up. She complained blood-tinged stool 1 month ago and elevated tumor marker was also found while following up. She came to 金門H. for help. Tumor over 20cm FAV was found in colonoscopy and malignancy was proved in biopsy. No distal organ metastasis was found in abdominal CT. Thus, she was transferred to our hospital and admitted for surgical intervention.

體檢發現

- (2021/01/27)
  Chest CT: GGN at LLL(#51), stable in size. Pneumonia patch over RUL with inflammatino process. Bil pleural effusion.

  (2019/10/02)
  Chest CT: GGN at LLL(#49). Some L't pleural effusion.

  (2018/08/15)
  Chest CT: GGO at LLL(#47). R't pleural effusion persist. Soft tissue mass over RLL.
  (2018/3/6)
  PET/CT:
  No tumor recurrence.
  (2016/10)
  SCC: 0.9 -> 0.6 -> 1.74 -> 0.6 -> 0.8 -> 1.2 -> 1.0 -> 0.5 -> 0.7
  CEA: 2.98 -> 1.78 -> 1.09 -> 1.62 -> 1.75 -> 2.19 -> 2.42 -> 2.38
  (2017/4)
  CT scan: GGO at LLL (#49, 3mm), stable in size. No recurrence. Pleural effusion stable.
  (2017/11)
  Chest CT: GGO at LLL (#47). R't pleural effusion persist. Soft tissue mass over RLL.

-

  Tumor maker: WNL.

  - General appearance :
    - easy looking , conscious clear , without cardiopulmonary distress
    - Vital signs自動帶入date 體溫(BT) 脈搏(P) 呼吸(R) 血壓(BP) 身高(BH) 體重(BW) BMI
      20211024 35.7℃ 93次/分 18次/分 123/91mmHg 162.5cm 40.2kg 15.2
  - HEENT :
    - Eyes: no anemic conjunctiva, no icteric sclera, normal EOM
    - ENT: no otorrhea, no rhinorrhea, no erythematous mucosa, no injected tonsil, no deformity
  - Neck: supple, freely movable, trachea midline,no deformity, no engorged jugular vein , no palpable thyroid or lymph node enlargement
  - Chest:
    - Inspection: Grossly normal, symmetric and fully expansion, no accessory muscle used, no operation scar, no spider angiomata, no gynecomastia
    - Palpation: no palpable mass or nodule, no subcutaneous emphysema, no tenderness
    - Ausculation: clear breathing sound
  - Heart:
    - Percussion: normal heart size
    - Ausculation: regular heart beat, normal S1, S2, no gallop, no murmur
  - Abdomen:
    - Inspection:
      - operation scar: +
    - Ausculation: normoactive bowel sound, no abdominal bruits
    - palpation: soft, no muscle guarding or rigidity, no local tenderness, no rebounding pain, no palpable mass, no palpable pulsation
    - Percussion: no shifting dulllness, no hepatomegaly, no splenomagaly
  -

Peripheral pulsation :	CA	BA	RA	FA	PA	PTA	DPA
Right side :	++	++	++	++	++	++	++/+++
Left side :	++	++	++	++	++	++	++/+++

  - Genito-anus: no tenderness or palpable mass lesion or rectal shelf, normal external genitalia, normal anal tone, brown stool

CRS CONDITION

- CEA: 3.4 ng/ml
-
  - Lung

手術日期及方法

- 手術日期及方法20211025 Thoracic intubation
  20211025 1. Intraoperative colonoscopy : (-)
  2. High ligation (+) : At IMA root
  3. take down splenic flexure (-)
  4. EndoGIA X1 + EEA (29mm) were used
  5. Cut margin: Proximal about 2.5 cm, distal about 5 cm
  6. A 12 Fr. Penrose drain was placed over the pelvic area

住院治療經過

- After admission, pigtail was inserted for left pleural effusion drainage and operation with laparoscopic anterior resection was done on 10/25. On POD1, hypotension was found so we adjusted PCA and gave low dose Levophed after survey. Levophed was turned off on POD3. We let her try diet under ERAS protocol. Pigtail was removed on 10/29. Under the stable condition, she was discharged on 10/30.

合併症

- nil

一般檢查

- 累積報告 :

DATE	TIME	ALB	TP	BIL.T	BIL.D	ALKP	AST	ALT	LDH	AMY	LIPASE	NH3	Ferritin	UA
1101025	1838	3.4		0.5				10						
1101028	0901	2.9		1.02	0.27		23	11						

DATE	TIME	WBC	RBC	HGB	HCT	MCV	PLT	BLST	NEUT	BAND	SEG	LYM	MON	EOS	BAS
1101025	1838	10220		11.2	34.9		185		88.4			7.5	3.3	0.4	0.4
1101026	1136			9.2											
1101028	0904	4130	4.58	12.6	39.9	87.1	156		77.2			10.9	7.3	4.1	0.5

DATE	TIME	NA	K	CL	BUN	CREAT	eGFR	CA	Mg	P	CRP	ESR	PCT	D-Dimer	LACTATE
1101025	1838	143	3.6		6	0.57		8.1							
1101026	1132														25.0
1101026	1137	141	3.7					8.0							
1101028	0901	140	3.6		6	0.70	92.68	7.3							

DATE	TIME	GLUCOSE	HbA1C	CHOL	TG	HDL	LDL	KETONE	CK	CKMB	TRO-T	PT-P	APTT-P	NT-pro BNP
1101026	1147									9				

DATE	TIME	PH	BEB	SCVO2	PO2	HCO3	HGB	PCO2	TCO2
1101026	1132	7.289	3.7	30.000	27.6	31.5		67.1	33.5

特殊檢查

病理報告

其它

出院時情況

- 3.改門診治療

出院指示

藥名	劑量	劑量單位	用法	頻次	數量	數量單位	現況	天數	單位
Flurbiprofen 100mg	1	TAB	PO	QD	11	TAB	TH	11	天
(口)Takepron OD tab 30mg	1	TAB	PO	QDAC	11	TAB	TH	11	天
10mg Singulair tab	1	TAB	PO	QN	11	TAB	TH	11	天
10mg Crestor tab	1	TAB	PO	QN	11	TAB	TH	11	天
Ultracet tab	1	TAB	PO	Q6H	44	TAB	TH	11	天
(口)Rosis tab 40mg	1	TAB	PO	QD	7	TAB	TH	7	天

出院計劃

主治醫師_2062F 蔣鋒帆 / 住院醫師_2331F 陳凱祥

正式摘要(電子簽章：**000000228516893)**

W83 / W83

doctorRank:B
